# *Staphylococcus aureus* nasal carriage in Ukraine: antibacterial resistance and virulence factor encoding genes

**DOI:** 10.1186/1471-2334-14-128

**Published:** 2014-03-05

**Authors:** Irina Netsvyetayeva, Mariusz Fraczek, Katarzyna Piskorska, Marlena Golas, Magdalena Sikora, Andrzej Mlynarczyk, Ewa Swoboda-Kopec, Wojciech Marusza, Beniamino Palmieri, Tommaso Iannitti

**Affiliations:** 1 Department of Medical Microbiology, Warsaw Medical University, Warsaw, Poland; 2 Chair and Department of General,Transplant and Liver Surgery, Warsaw Medical University, Warsaw, Poland; 3 Department of Dentistry Microbiology, Warsaw Medical University, Warsaw, Poland; 4 Academy of Face Sculpturing, Warsaw, Poland; 5 Department of General Surgery and Surgical Specialties, University of Modena and Reggio Emilia Medical School, Surgical Clinic, Modena, Italy; 6 School of Biomedical Sciences, University of Leeds, Mount Preston Street, Garstang building, LS2 9JT Leeds, UK

**Keywords:** *Staphylococcus aureus*, Methicillin Sensitive *S. aureus*, Methicillin Resistant *S. aureus*, Panton-Valentine Leukocidin, Exfoliative Toxins

## Abstract

**Background:**

The number of studies regarding the incidence of multidrug resistant strains and distribution of genes encoding virulence factors, which have colonized the post-Soviet states, is considerably limited. The aim of the study was (1) to assess the *Staphylococcus (S.) aureus* nasal carriage rate, including Methicillin Resistant *S. aureus* (MRSA) strains in adult Ukrainian population, (2) to determine antibiotic resistant pattern and (3) the occurrence of Panton Valentine Leukocidine (PVL)-, Fibronectin-Binding Protein A (FnBPA)- and Exfoliative Toxin (ET)-encoding genes.

**Methods:**

Nasal samples for *S. aureus* culture were obtained from 245 adults. The susceptibility pattern for several classes of antibiotics was determined by disk diffusion method according to the European Committee on Antimicrobial Susceptibility Testing (EUCAST) guidelines. The virulence factor encoding genes, *mecA, lukS-lukF, eta, etb, etd, fnbA,* were detected by Polymerase Chain Reaction (PCR).

**Results:**

The *S. aureus* nasal carriage rate was 40%. The prevalence of nasal MRSA carriage in adults was 3.7%. *LukS-lukF* genes were detected in over 58% of the strains. ET-encoding genes were detected in over 39% of the strains and the most prevalent was *etd*. The *fnbA* gene was detected in over 59% of the strains. All MRSA isolates tested were positive for the *mecA* gene. *LukS-lukF* genes and the *etd* gene were commonly co-present in MRSA*,* while *lukS-lukF* genes and the *fnbA* gene were commonly co-present in Methicillin Sensitive *S. aureus* (MSSA) isolates. No significant difference was detected between the occurrence of *lukS-lukF* genes (P > 0.05) and the *etd* gene (P > 0.05) when comparing MRSA and MSSA. The occurrence of the *fnbA* gene was significantly more frequent in MSSA strains (P < 0.05)*.*

**Conclusions:**

In Ukraine, *S. aureus* is a common cause of infection. The prevalence of *S. aureus* nasal carriage in our cohort of patients from Ukraine was 40.4%. We found that 9.1% of the strains were classified as MRSA and all MRSA isolates tested positive for the *mecA* gene. We also observed a high prevalence of PVL- and ET- encoding genes among *S. aureus* nasal carriage strains. A systematic surveillance system can help prevent transmission and spread of drug resistant toxin producing *S. aureus* strains.

## Background

Nasal colonization is the cause of recurrent staphylococcal infections. The host tissue colonization by *Staphylococcus (S.) aureus* is an important factor in disease pathogenesis. *S. aureus* expresses Fibronectin-Binding Protein A (FnBPA), which mediates the adhesion to fibrinogen, elastin and fibronectin [1]. The pathogenicity of *S. aureus* results from its ability to produce specific toxins and hydrolytic enzymes. Some *S. aureus* strains can produce Panton-Valentine Leucocidin (PVL), which can cause tissue necrosis and leukocyte lysis [2]. PVL is a bi-component leucotoxin composed of S-related and F-related proteins that are separately secreted, but act synergistically. The Exfoliative Toxins (ET) may cause the staphylococcal scalded-skin syndrome, Ritter disease, and bullous impetigo [3]. Three serological forms of ET, i.e. ETA, ETB and ETD have been linked to human pathologies [4]. The investigation into *S. aureus* antimicrobial susceptibility pattern presents a research question for national public health programs in Europe and other countries [5-9]. Increasing resistance to antibiotics and the growing prevalence of Methicillin Resistant *S. aureus* (MRSA) can be connected to antibiotic overuse in primary care and requires to be addressed promptly [10,11]. Ukraine is not part of the European Union (EU), but it borders several EU countries. Ukrainian doctors have limited access to international information and do not take part in European research programs on epidemiology or bacterial drug resistance. Up to now, there has been only a report in 2009 on the antimicrobial susceptibility pattern of *S. aureus,* cultured from clinical samples in 97 surgical wards located in Ukraine [12]. In this study, the prevalence of MRSA ranged from 10.8% to 84.1% depending on the location. The authors reported that it was not possible to evaluate the general consumption of antibiotics outside the hospital environment, since they could be purchased without medical prescription. The aim of our study was (1) to assess the *S. aureus* nasal carriage rate including MRSA strains in adult Ukrainian population, (2) to determine the antibiotic resistant pattern and (3) the occurrence of PVL-, FnBPA- and ET-encoding genes.

## Methods

### Patients

Samples were obtained in the South-West Ukraine from a medical mission organized by the Polish Community Association in July 2011. Nasal swabs were obtained from inhabitants of small towns and communities coming for medical consultations. Samples were collected from both anterior nares by rotating a sterile Amies agar gel medium transport swab (Oxoid, Basingstoke, UK) and they were sent to the Department of Clinical Microbiology at the Warsaw Medical University (Warsaw, Poland). Only one isolate from each patient was included in the study. Each patient completed a standardized questionnaire including his age, gender, and medical history. All patients signed the informed consent. This study was planned and performed in accordance with the Helsinki Declaration and was approved by the Ethics Committee of the Polish Community Association. Inclusion criterion was: age ≥ 18 years. Exclusion criteria were: infections, use of antibiotics and hospitalization in the three months preceding the beginning of the study.

### *S. aureus* isolation and identification

Nasal swabs were inoculated directly onto 2 ml of Brain Heart Infusion (BHI) liquid broth (bioMerieux, Marcy l’Etoile, France). Samples were incubated for 24 h at 37°C, then inoculated onto blood agar (Oxoid, Basingstoke, UK), Chapmann agar (Oxoid, Basingstoke, UK) and finally onto selective MRSA agar plates (Oxoid, Basingstoke, UK), which were then incubated for 24–48 h at 37°C. Identification of *S. aureus* isolates was completed with traditional biochemical tests, including Gram-staining, haemolysis, mannitol fermentation, and the latex slide agglutination test (Staphytect Plus; Oxoid, Basingstoke, UK). Ambiguous strains were additionally identified with the Api ID32 Staph test (bioMerieux, Marcy l’Etoile, France) and the results were read using the ATB-expression system (bioMerieux, Marcy l’Etoile, France).

### Susceptibility testing

Antibiotic susceptibility testing was performed by disk diffusion method following the European Committee on Antimicrobial Susceptibility Testing (EUCAST) guidelines. The tested antimicrobial agents were: cefoxitin (30 μg), gentamicin (10 μg), tetracycline (30 μg), trimethoprim-sulfamethoxazole (1.25/23.75 μg), erythromycin (15 μg), clindamycin (2 μg), ciprofloxacin (5 μg), mupirocin (200 μg), rifampicin (5 μg), fusidic acid (10 μg) and penicillin (1 μg) (all Oxoid, Basingstoke, UK). Isolates were classified as susceptible or resistant based on *S. aureus* epidemiological cut-off values issued by the EUCAST. Erythromycin-induced clindamycin resistance was detected by Disk approximation test (D-test). The reference strain *S. aureus* ATCC 29213 was used as internal quality control. The nitrocefine test was completed using beta-lactamase identification sticks (Oxoid, Basingstoke, UK). The sizes of inhibition zone diameters were independently read by at least three operators and then averaged to obtain the final inhibition zone diameters (in mm).

### Polymerase Chain Reaction (PCR)

DNA was extracted using the Genomic DNA Extraction kit (EURx, Gdansk, Poland) according to the manufacturer’s guidelines. The cefoxitin-resistant isolates were analyzed for the presence of the *mecA* gene using PCR, as previously described [13]. PCR, used to detect PVL-, FnBPA-, and ET-encoding genes, was performed as previously described [14-17]. Primers and conditions for PCR amplification used in this study are listed in Table 1.

**Table 1:** Primers used for detection of PVL-, FnBPA-, and ET-encoding genes by PCR

**Gene**	**Primer**	**Primer sequence (5’ – 3’)**	**Amplicon size (bp)**	**Reference**
*mecA*	*mecA*-F	GTAGAAATGACTGAACGTCCGATAA	310	McClure et al. 2006 [13]
	*mecA*-R	CCAATTCCACATTGTTTCGGTCTAA		
*fnbA*	*fnbA*-F	CACAACCAGCAAATATAG	1362	Peacock et al. 2002 [14]
	*fnbA*-R	CTGTGTGGTAATCAATGTC		
*eta*	*eta*-F	ACTGTAGGAGCTAGTGCATTTGT	190	Jarraud et al. 2002 [15]
	*eta*-R	TGGATACTTTTGTCTATCTTTTTCATCAAC		
*etb*	*etb*-F	ATACACACATTACGGATAAT	629	Yamaguchi et al. 2001 [16]
	*etb*-R	CAAAGTGTCTCCAAAAGTAT		
*etd*	*etd* - F	CGCAAATACATATGAAGAATCTGA	452	Nakaminami et al. 2008 [17]
	*etd* - R	TGTCACCTTGTTGCAAATCTATAG		
PVL components S and F	*lukS*-PV-	AGTGAACTTATCTTTCTATTGAAAAACACTC	433	Jarraud et al. 2002 [15]
	*lukF*-PV	GCATCAASTGTATTGGATAGCAAAAGC		

### Statistical analysis

Pearson’s, and Yates’ chi-square tests were used to assess inter-group significance. Statistical significance was assumed at P < 0.05. Statistical analysis was completed using Statistica Software version 5.0 (STAT Soft, Cracow, Poland).

## Results

Ninety nine strains of *S. aureus* were isolated from nasal swabs taken from 245 adults (184 females, and 61 males). The mean female age was 58.2 ± 17.6 (range: 20–91). The mean male age was 53.5 ± 18.8 (range: 18–82). The prevalence of *S. aureus* nasal carriage was 40.4% (99/245). Nine out of 99 (9.1%) strains were classified as MRSA by using the cefoxitin disk diffusion method. All MRSA isolates tested positive for the *mecA* gene. The prevalence of nasal MRSA was 3.7% (9/245). Antimicrobial susceptibility profiles and distribution of virulence factor encoding genes of *S. aureus* nasal carriage isolates are presented in Table 2.

**Table 2:** ***Staphylococcus aureus *** **nasal carriage isolates: antimicrobial susceptibility profiles and distribution of virulence factor encoding genes**

		**ANTIMICROBIAL**												**VIRULENCE FACTOR ENCODING GENES**				
***S. aureus***	**Nitrocefine test**	**Gentamicyn**	**Mupirocin**	**Rifampicin**	**Tetracyclin**	**Ciprofloxacin**	**Sulphamethoxazole/trimethoprim**	**Penicilin G**	**Fusidic acid**	**Cefoxitin**	**Clindamycin**	**Erythromycin**	**MLSB**	***luk*** **PV**	***eta***	***etb***	***etd***	***fnbA***
**MSSA**																		
1		S	S	S	S	S	S	S	S	S	S	S					+	
2	+	S	S	S	S	S	S	R	S	S	S	S						
3	+	S	S	S	S	S	S	R	S	S	S	S		+				+
4	+	S	S	S	S	S	S	R	S	S	S	S						
5	+	R	S	R	R	S	S	R	S	S	R	R		+				
6	+	S	S	S	S	S	S	R	S	S	S	S		+		+		+
7	+	S	S	S	S	S	S	R	S	S	S	S		+			+	+
8	+	S	S	S	S	S	S	R	S	S	S	S					+	+
9	+	S	S	S	S	S	S	R	S	S	S	S		+			+	+
10	+	S	S	S	S	S	S	R	S	S	S	S		+				+
11	+	S	I	R	S	S	S	R	S	S	S	S						+
12		S	S	S	S	S	S	S	S	S	S	S		+				
13	+	S	S	S	S	S	S	R	S	S	S	S		+				+
14	+	S	S	S	S	S	S	R	S	S	S	S		+				+
15	+	S	S	S	S	S	S	R	S	S	S	R	+	+		+		+
16	+	S	S	S	S	S	S	R	S	S	S	S		+			+	+
17	+	R	S	R	S	S	I	R	S	S	R	R		+			+	+
18	+	S	S	S	S	S	S	R	S	S	S	S		+				+
19		S	S	S	S	S	S	S	S	S	S	S		+				+
20		S	S	S	S	S	S	S	S	S	S	S						
21	+	S	S	S	S	S	S	R	S	S	S	S		+			+	+
22	+	S	S	S	S	S	S	R	S	S	S	S		+				+
23	+	R	S	S	R	S	S	R	S	S	S	R	+	+				+
24	+	S	S	S	S	S	S	R	S	S	S	S						
25		S	S	S	S	S	S	S	S	S	S	S				+		
26		S	S	S	S	S	S	S	S	S	S	S		+				
27	+	S	S	S	S	S	S	R	S	S	S	S		+			+	+
28	+	S	I	S	S	S	S	R	S	S	S	S		+				+
29	+	S	S	S	R	S	S	R	S	S	S	S						
30		S	S	S	S	S	S	S	S	S	S	S				+		+
31	+	S	S	S	S	S	S	R	S	S	S	S						
32		S	S	S	S	S	S	R	S	S	S	S		+				+
33		S	S	S	S	S	S	S	S	S	S	S		+				+
34	+	S	S	S	S	S	S	R	S	S	S	R		+			+	+
35	+	S	S	S	S	S	S	R	S	S	S	S		+				+
36	+	S	I	S	R	S	S	R	S	S	S	S		+				
37	+	S	S	S	S	S	S	R	S	S	S	S						
38	+	S	S	S	S	S	S	R	S	S	S	S						+
39	+	S	S	S	S	S	S	R	S	S	S	S		+				+
40		S	S	S	S	S	S	S	S	S	S	S		+			+	+
41	+	S	S	S	S	S	S	R	S	S	S	S		+				
42		S	S	R	S	S	S	S	S	S	S	S						
43	+	S	S	S	S	S	S	R	R	S	S	S		+		+		+
44	+	S	S	S	S	S	S	R	S	S	S	S		+				+
45	+	S	S	S	S	S	S	R	S	S	S	S		+		+		
46	+	S	S	S	S	S	S	R	S	S	S	S				+		+
47	+	S	S	S	S	S	S	R	S	S	S	S		+				
48	+	S	S	S	S	S	S	R	S	S	S	S						
49	+	S	S	S	S	S	S	R	S	S	S	S		+			+	+
50	+	R	S	S	S	S	S	R	R	S	S	R	+	+				+
51	+	S	S	S	S	S	S	R	S	S	S	S		+				+
52		S	S	S	S	S	S	S	S	S	S	S		+				+
53	+	S	S	S	S	S	S	R	S	S	S	S		+				
54	+	S	S	S	S	S	S	R	S	S	S	S						+
55	+	S	S	S	S	S	S	R	S	S	S	S		+				+
56	+	S	S	S	S	S	S	R	S	S	S	S						+
57	+	S	S	S	S	S	S	R	R	S	R	S			+		+	+
58		S	I	S	R	S	S	S	S	S	S	S		+			+	+
59	+	S	S	S	S	S	S	R	S	S	S	S		+	+			+
60	+	S	S	S	S	S	S	R	S	S	S	S						
61	+	S	S	S	S	S	S	R	S	S	S	S					+	+
62	+	S	S	S	S	S	S	R	S	S	S	S			+		+	+
63	+	S	S	S	S	S	S	R	S	S	S	S		+				+
64	+	S	S	S	S	S	S	R	S	S	S	S		+				+
65		S	S	S	S	S	S	S	S	S	S	S						
66	+	S	S	S	S	S	S	R	S	S	S	S		+				
67	+	S	S	S	S	S	S	R	S	S	S	S		+			+	+
68	+	S	S	S	S	S	S	R	S	S	S	S						
69		S	S	S	S	S	S	S	S	S	S	S						
70		S	S	S	S	S	S	S	S	S	S	S		+				+
71	+	S	S	S	S	S	S	R	R	S	S	S		+		+		+
72	+	S	S	S	S	S	S	R	S	S	S	S			+			
73	+	S	S	S	S	S	S	R	S	S	S	S						
74	+	S	S	S	S	S	S	R	S	S	S	S		+			+	+
75	+	S	S	S	S	S	S	R	S	S	S	S		+				+
76	+	S	S	S	S	S	S	R	S	S	S	S		+				+
77		S	S	S	S	S	S	S	S	S	S	S		+				+
78		S	S	S	S	S	S	R	S	S	S	S					+	+
79		S	S	S	S	S	S	S	S	S	S	S						+
80	+	S	S	S	S	S	S	R	S	S	S	S					+	+
81	+	S	S	S	S	S	S	R	R	S	S	S		+			+	+
82		S	I	S	S	S	S	S	S	S	S	S		+				
83		S	S	S	S	S	S	S	S	S	S	R	+					
84	+	S	S	S	S	S	S	R	S	S	S	S						+
85		S	S	S	S	S	S	S	S	S	S	S		+				+
86	+	R	S	S	S	S	R	R	R	S	S	R	+				+	
87	+	S	S	S	S	S	S	R	S	S	S	S						
88	+	S	S	S	S	S	S	R	S	S	S	S						
89	+	S	S	S	S	S	S	R	S	S	S	S						
90		S	I	S	R	S	S	R	S	S	R	S						+
**MRSA**																		
1	+	S	S	S	R	S	S	R	R	R	S	R	+	+				
2		R	I	S	R	S	I	R	R	R	R	R						
3	+	S	S	R	R	S	S	R	R	R	S	R		+			+	
4	+	R	I	I	R	S	R	R	R	R	R	R						
5	+	S	S	S	R	S	S	R	R	R	S	R	+	+			+	
6	+	R	I	R	S	S	S	R	R	R	R	S				+	+	
7		S	S	S	R	S	S	R	R	R	S	R	+	+			+	
8	+	S	S	S	S	S	S	R	R	R	S	S		+		+		+
9	+	R	I	R	R	S	S	R	S	R	R	R						

S – susceptibility, R – resistance, I – intermediate susceptibility, MLSb – erythromycin induced clindamycin resistance.

### Antibiotic susceptibility pattern

All *S. aureus* strains were susceptible to ciprofloxacin. No ciprofloxacin- or mupirocin-resistant strains were detected, but, as far as the level of antimicrobial agent resistance to mupirocin was concerned, 10.1% (10/99) of the strains were classified as intermediate. The ability to produce beta-lactamases was detected in 74.7% (74/99) of the strains. 77.4% (67/90) of MSSA and 77.8% (7/9) of MRSA strains resulted positive in the nitrocefin test. MSSA and MRSA displayed no statistical difference in beta-lactamase secretion (P > 0.05). 77.8% (70/90) of MSSA isolates were resistant to penicillin. Resistance to erythromycin was observed in 10% (9/90), to fusidic acid and tetracycline in 6.7% (6/90), to gentamicin in 5.5% (5/90), to clindamycin and rifampicin in 4.4% (4/90), and to trimethoprim-sulfamethoxazole in 1.1% (1/90) of MSSA isolates. All MRSA strains were ciprofloxacin-susceptible. Seven out of 9 (77.8%) MRSA strains were trimethoprim/sulfamethoxazole-susceptible. Five out of 9 (55.6%) were susceptible to mupirocin, rifampicin, gentamicin. Eight strains showed resistance to fusidic acid. 77.8% of those strains presented resistance to tetracycline, erythromycin, and clindamycin (7 out of 9, 7 out of 9, and 4 out of 9, respectively). Erythromycin-induced clindamycin resistance occurred in 3 out of 9 (33.3%) MRSA strains and in 5 out of 90 (5.6%) MSSA strains. The erythromycin-induced clindamycin resistance rate was significantly higher among MRSA strains, if compared with MSSA strains (P < 0.05).

### Distribution of virulence factor encoding genes

The virulence factor encoding genes were detected in 79.8% (79/99) of the isolates. Among the 99 strains, the *fnbA* gene was detected in over 59% of the strains, *lukS-lukF* genes in over 58% and ET-encoding genes in over 39%. The *lukS-lukF* genes were detected in 55.5% (5/9) of MRSA and in 58.9% (53/90) of MSSA strains. *LukS-lukF* genes and the *etd* gene were most commonly co-present in MRSA strains, whereas *lukS-lukF* genes and the *fnbA* gene were most commonly co-present in MSSA strains. No significant difference was detected between the occurrence of *lukS-lukF* genes (P > 0.05) and the *etd* gene (P > 0.05), whereas the occurrence of the *fnbA* gene was significantly more frequent in MSSA strains (P < 0.05)*.*

## Discussion

There is no evidence of *S. aureus* susceptibility and occurrence of virulence encoding genes within the Ukrainian population. The present study reports that the prevalence of *S. aureus* nasal carriage in Ukraine was 40.4%, whereas MRSA carriage was 3.7%. In general, *S. aureus* nasal carriage rate in Ukraine is higher than in most countries in Europe, Africa, Asia, North and South America and Oceania [5-7,18-23]. In our study, neither MSSA nor MRSA ciprofloxacin resistant strains were observed using the disk diffusion method. These data differ from the data concerning other countries [5,7,18,23]. The absence of ciprofloxacin resistant strains can be explained by their limited use in Ukraine. In this study the ET-encoding genes were found in about 40% of *S. aureus* strains. The most prevalent serotype was ETD. Our data differ from the data concerning other European, American, and African countries, where the ETA serotype is prevalent and detected in more than 80% of toxin-producing strains [24-26]. Only in Japan, ETB-producing strains are more prevalent than those expressing ETA [27]. In this study, no significant difference was detected in the occurrence of ET-encoding genes between MRSA and MSSA. Other studies have suggested that the *etb* gene was found primarily in strains with *mecA*, while the *eta* gene was mainly found in strains without *mecA*[15]. The prevalence of *fnbA*-positive *S. aureus* in healthy Ukrainian adults is lower, if compared to its prevalence in other countries [28]. In the present study, FnBPA-encoding gene was detected in over 59% of strains, whereas the occurrence of the *fnbA* gene was more frequent in the MSSA*.* We found a high prevalence of PVL encoding genes. Over 58% strains isolated from nares in individuals with no staphylococcal infection symptoms, were *luk*-PV-positive. This evidence contrasts with previous reports. For instance, the prevalence of PVL-positive *S. aureus* nasal colonization in Dutch general practice patients was 0.6% [5]. Furthermore, a PVL prevalence of 38.9% was observed in *S. aureus* and it caused abscesses, arthritis, and soft-tissue infections [5]. The prevalence of PVL-positive *S. aureus* in nasal colonization was 2.4% in the United States. The data were obtained as part of the National Health and Nutrition Examination Survey [6]. It was estimated that PVL-positive *S. aureus* was more prevalent pathogen in the tropics and subtropics, if compared with European countries [29]. The PVL genes were detected in 10.6% methicillin sensitive *S. aureus* strains in the Indonesian population [30]. A similar PVL-positive percentage (around 57%) was discovered in an African study of five cities in Cameroon, Morocco, Madagascar, Niger and Senegal. However, the tested group consisted of individuals with an already diagnosed staphylococcal infection [31]. Therefore, the PVL-positive percentage in the Ukrainians with staphylococcal infection symptoms would be significantly higher. Travellers to tropical and subtropical countries are exposed to a higher risk of skin and soft-tissue infections. This phenomenon results from a higher PVL-positive *S. aureus* occurrence in tropical and subtropical countries [31]. Similarly, job seekers travelling from Ukraine could be a source of toxin-producing strains.

## Conclusions

In our cohort of Ukrainian patients, we found that the prevalence of *S. aureus* nasal carriage was 40.4%. 9.1% of the strains were classified as MRSA and all MRSA isolates tested positive for the *mecA* gene. The prevalence of nasal MRSA was 3.7%. We also found a high prevalence of PVL- and ET-encoding genes among *S. aureus* nasal carriage strains. A limitation of our study is that we studied isolates deriving only from the South-West Ukraine and it cannot be representative of the overall Ukrainian situation. Furthermore, we did not perform *spa* typing and therefore we could not discriminate among different strains of *S. aureus*. Further studies are required to address those limitations. A systematic surveillance system can help prevent transmission and spread of drug resistant toxin producing *S. aureus* strains.

## Competing interests

The authors declare that they have no competing interests.

## Authors’ contributions

IN designed the study, collected samples and data, performed microbiological and molecular testing and data analysis, interpreted the results, participated in the drafting of the article and obtained funding; MF designed and coordinated the study, collected samples, interpreted the results, analysed the data and obtained funding; KP collected data, performed microbiological and molecular testing, participated in the drafting of the article and obtained funding; MG collected data, performed microbiological and molecular tests and participated in the drafting of the article; MS collected data, performed microbiological and molecular tests and participated in the drafting of the article; AM analyzed the data and participated in the drafting of the article; ES-K coordinated the study and performed data analysis; WM performed data analysis and coordinated the study; BP designed and coordinated the study; TI supervised and coordinated the study, performed data analysis and participated in the drafting of the article. All authors read and approved the final version of the manuscript.

## Pre-publication history

The pre-publication history for this paper can be accessed here:

http://www.biomedcentral.com/1471-2334/14/128/prepub
